# Regulatory Effects and Mechanisms of Electromagnetic Low-Energy Shock Wave Therapy on Blood Glucose in Type 2 Diabetic Rats

**DOI:** 10.3390/biology15100810

**Published:** 2026-05-20

**Authors:** Yinghui He, Shuying Huo, Linyao Hao, Zipeng Yue, Ying Jiang, Jianhua Zhu, Zhidong Zhou

**Affiliations:** College of Veterinary Medicine, Hebei Agricultural University, Baoding 071001, China; heyinghui97@163.com (Y.H.); 15231928521@163.com (L.H.); 17340921526@163.com (Z.Y.); 18831272392@163.com (Y.J.);

**Keywords:** electromagnetic low-energy shock wave, type 2 diabetes mellitus, glucose metabolism, pyroptosis pathways

## Abstract

The global incidence of type 2 diabetes mellitus is rising rapidly, yet current management strategies remain largely supportive. Electromagnetic low-energy shock wave (ELESW) therapy, known for its anti-inflammatory and pro-angiogenic properties, is clinically used for lithotripsy and pain management. This study aimed to investigate the potential of ELESW to regulate blood glucose and to explore its underlying mechanisms in T2DM rats. To determine safe treatment parameters, a T2DM model was first established in Sprague–Dawley (SD) rats using a high-fat, high-sugar diet combined with low-dose streptozotocin injection. Therapeutic efficacy was then assessed in obese OLETF rats, a spontaneous T2DM model, by monitoring body weight, fasting and postprandial blood glucose, glucose tolerance, insulin tolerance, and histopathology. To explore the mechanisms, insulin-resistant (IR-HepG2) and high-glucose-damaged (HG-HepG2) human hepatocyte models were used to evaluate the effects of ELESW on glucose metabolism and pyroptosis pathways. Safe ELESW parameters were established as <7 kV and <200 pulses. In T2DM OLETF rats, treatment (7 kV, 150 pulses) significantly improved glycemic control and insulin sensitivity, alleviated systemic insulin resistance, promoted islet repair, suppressed inflammation, and reduced renal injury. In vitro, ELESW altered mRNA levels of glucose metabolism genes (e.g., PI3K/AKT, AMPK) and genes related to proliferation, pyroptosis, and apoptosis. These transcriptional changes suggest that ELESW may enhance glucose uptake and improve β-cell function, possibly through PI3K/AKT and AMPK signaling; however, protein-level confirmation is needed. These findings suggest that ELESW ameliorates hyperglycemia in T2DM rats, warranting further investigation as a potential non-pharmacological intervention.

## Simple Summary

The global incidence of type 2 diabetes mellitus is rising rapidly, and current drug-based treatments often have side effects and cannot halt disease progression. This study explored a non-invasive physical therapy—electromagnetic low-energy shock wave (ELESW)—as a potential approach to regulate blood glucose in diabetic rats. First, safe ELESW parameters were identified (voltage < 7 kV, pulses < 200). Then, T2DM rats were treated with ELESW at 7 kV and 150 pulses. The results showed that ELESW significantly lowered blood glucose; improved insulin resistance; reduced inflammation in the pancreas, liver, and kidneys; and promoted the repair of insulin-producing β-cells. In cell experiments, ELESW affected the expression of genes involved in glucose metabolism (e.g., PI3K/AKT and AMPK pathways) and was simultaneously associated with reduced expression of genes related to cell pyroptosis and apoptosis. These findings suggest that ELESW may represent a preclinical candidate for a non-pharmaceutical complementary approach to managing type 2 diabetes, but further validation is required.

## 1. Introduction

Diabetes mellitus is a metabolic disorder characterized by chronic hyperglycemia, primarily resulting from insufficient insulin secretion and insulin resistance [1]. As a core pathological feature of type 2 diabetes mellitus (T2DM), insulin resistance manifests as decreased tissue sensitivity to insulin, coupled with progressive failure of pancreatic β-cells to compensate, ultimately leading to a chronic hyperglycemic state [2,3]. This state, in turn, triggers a cascade of pathological events, including β-cell apoptosis [4], dysregulation of glucose and lipid metabolism [5], oxidative stress, and inflammatory responses [6,7]. The central goals in managing this complex disease are effective glycemic control, amelioration of insulin resistance, and preservation of β-cell function. While pharmacological interventions remain the mainstay of treatment, they are often associated with side effects and an inability to halt disease progression [8]. Therefore, exploring novel, non-invasive, and potentially multi-targeted alternative or adjuvant therapies is of paramount importance.

In recent years, mechanical wave-based physical therapies have gained increasing attention in diabetes and complications research. One study demonstrated that hepatic focused ultrasound alleviated obesity and related complications in mice, significantly reducing body weight, blood lipids, and lipoprotein dysregulation, while also decreasing hepatic cytokine levels and leukocyte infiltration [9]. Subsequent research showed that targeted hepatic focused ultrasound stimulation of the hepatoportal nerve plexus markedly improved glucose metabolism in diabetic mice, rats, and pigs, thereby ameliorating insulin resistance and treating T2DM. It is proposed that the mechanical waves generated by ultrasonic pulses act by activating mechanosensitive ion channels and modulating peripheral nerves. This approach has completed in vitro and preclinical model studies and has now entered phase I clinical trials [10,11]. Meanwhile, electromagnetic low-energy shock wave (ELESW) therapy, a non-invasive physical treatment, has demonstrated effects in orthopedics and urology, including promoting tissue regeneration and angiogenesis and inhibiting inflammation [12,13]. In recent years, numerous studies have reported its efficacy in wound healing and in treating diabetes and its complications [14,15,16,17]. Preliminary evidence suggests that low-energy shock waves can regulate local blood flow and may influence specific cellular signaling pathways [18,19], and may even promote the regeneration of damaged pancreatic β-cells [20], providing a rationale for their application in diabetes treatment.

To date, whether ELESW therapy directly improves systemic glucose regulation in T2DM has not been systematically investigated. Previous studies have focused primarily on the effects of shock waves on wound healing, local blood flow, or tissue regeneration in diabetic complications, rather than on pancreatic β-cell function or hepatic glucose metabolism. Furthermore, the safety profile of ELESW in the context of diabetic vascular fragility remains unexplored. To address these gaps, the present study first established a T2DM model in SD rats to identify safe ELESW treatment parameters and to characterize the potential hazards associated with excessive energy delivery. Subsequently, therapeutic efficacy was evaluated in spontaneously diabetic OLETF rats, a model that closely recapitulates the progressive nature of human T2DM. In addition, ELESW was applied to IR-HepG2 and HG-HepG2 hepatocytes to explore its effects on hepatic glucose metabolism at the cellular level. This integrated approach aims to investigate whether ELESW can ameliorate insulin resistance, protect pancreatic β-cells, and alleviate hyperglycemia-induced hepatocyte damage, providing a preliminary basis for further evaluation of ELESW as a potential non-pharmacological intervention in T2DM.

## 2. Materials and Methods

### 2.1. Experimental Animals and Cells

Animals: Sprague–Dawley (SD) rats were purchased from Beijing SPF Biotechnology Co., Ltd. (Beijing, China). Obese Otsuka Long-Evans Tokushima Fatty (OLETF) rats were obtained from Beijing HFK Bioscience Co., Ltd. (Beijing, China). All rats were housed individually in a standard specific pathogen-free (SPF) animal facility with free access to food and water. The environment was maintained at a temperature of 22 ± 2 °C, humidity of 50 ± 10%, and a 12-h light/12-h dark cycle. The experimental protocol was reviewed and approved by the Animal Ethics Committee of Hebei Agricultural University (Approval No. 2023200). The approval specifically covered the observation of severe adverse events, including hemorrhage and mortality, with predefined humane endpoints (e.g., >20% body weight loss within one week, inability to eat or drink, or moribund state). Animals reaching these endpoints were immediately euthanized by intraperitoneal injection of sodium pentobarbital (150 mg/kg). All procedures were performed in accordance with the principles of animal welfare and ethics.

Cell lines: The HepG2 cell line (human hepatocellular carcinoma cell line) was purchased from Shanghai Fuheng Biotechnology Co., Ltd. (Shanghai, China).

### 2.2. Drugs and Reagents

Streptozotocin (Bio-Target, Beijing, China, Catalog No. S6050F). Isoflurane (Jindafu Pharmaceutical, Xingtai, China; Lot No. 2022504). Anhydrous glucose (TOPBIO, Beijing, China, Catalog No. 0188-500 g). Insulin (Novo Nordisk, Tianjin, China, Lot No. 2022083642). DMEM medium (Gibco, Suzhou, China, Lot No. 6125109). Penicillin–streptomycin solution (Wisent, Nanjing, China, Catalog No. 450-201-E1). Fetal bovine serum (Servicebio, Wuhan, China, Catalog No. G8003-100 mL). MTT (Solarbio, Beijing, China, Catalog No. M8180). DMSO (Servicebio, Wuhan, China, Catalog No. GC203006-100 mL). Animal Tissue/Cell Total RNA Extraction Kit (Servicebio, Wuhan, China, Catalog No. G2640-50T). Reverse Transcription Kit (Servicebio, Wuhan, China, Catalog No. G3337-50). SYBR Green qPCR Master Mix (Servicebio, Wuhan, China, Catalog No. G3326-01). Primer design and synthesis (Servicebio, Wuhan, China). Rat Insulin ELISA Kit (Yuanju Bio, Shanghai, China, Lot No. 202404). ALT ELISA Kit (Nanjing Jiancheng, Nanjing, China, Catalog No. C009-1). AST Assay Kit (Nanjing Jiancheng, Nanjing, China, Catalog No. C010-1). Glycogen Assay Kit (Nanjing Jiancheng, Nanjing, China, Catalog No. A043-1-1).

### 2.3. Equipment and Instruments

The electromagnetic low-energy shock wave experimental device used in this study was a modified KDE-2B electromagnetic shock wave lithotripter (energy range: 6–40 J; voltage: ~220 V; frequency: 50 Hz; input power: 1.5 kVA), which was jointly developed by the research teams from Hebei Agricultural University and Beijing Zhongke Jian’an Medical Technology Co., Ltd. (Hebei, China). The device incorporates a focusing lens and a dual-layer damping interface. The focal zone has a diameter of approximately 15 mm and a length of approximately 25 mm, located at a depth of 10–15 mm below the skin surface. The energy flux density was set to <0.08 mJ/mm^2^. The treatment area (liver and pancreas projection) was defined as the region between the xiphoid process and the left costal margin. To ensure reproducibility, the probe was manually positioned using anatomical landmarks (xiphoid process and the lowest point of the left rib cage), with the same operator performing all treatments. Medical ultrasound coupling gel was applied consistently at a thickness of 2–3 mm, with the probe maintained perpendicular to the skin surface. The same energy dose was delivered to both liver and pancreas regions by sequentially targeting the two areas during each session; no repositioning was allowed during pulse delivery. All treatments were performed by a single operator who was aware of group allocation. On Call EZ IV Blood Glucose Meter (ACON Biotechnology (Hangzhou) Co., Ltd.). (Hangzhou, China). Cell culture incubator (MCO-15AC, SANYO Electric Biomedical Co., Ltd.). (Moriguchi, Japan). Microplate reader (iMark, purchased from BIO-RAD). (Hercules, CA, USA). CFX Connect Optics Module q-PCR instrument (purchased from BIO-RAD). DP-73 optical microscope (purchased from OLYMPUS). (Tokyo, Japan).

### 2.4. Establishment and Grouping of the T2DM Model in SD Rats

SD rats were divided into a normal group and a model group for the evaluation of the efficacy and safety of shock wave parameters. Rats in the model group were fed a high-fat and high-sugar diet (formula: 20% glucose, 10% animal oil, 0.5% sodium cholate, and 1% cholesterol) for 12 weeks, followed by intraperitoneal injection of streptozotocin (STZ, 30 mg/kg) for three consecutive days. Before STZ injection, the rats were fasted for 12 h with free access to water. All rats except those in the normal group received STZ dissolved in citrate buffer; the normal group received citrate buffer only. Three days after the last injection, blood glucose levels were measured by tail puncture using a glucometer. Only rats with blood glucose levels above 11.1 mmol/L were included in the subsequent grouping experiments. The SD rat model was chosen for parameter optimization due to its rapid and predictable induction of T2DM, which facilitates the efficient identification of lethal and safe energy thresholds with minimal animal use (3R principle).

Rats were anesthetized with 2.0% isoflurane by inhalation, placed in lateral recumbency on an animal board, and fixed. The hair over the liver and pancreas in the abdominal region was shaved, and medical ultrasound coupling gel was applied to the exposed skin. The shock wave probe was placed over the liver and pancreas. Shock wave parameters were as follows: output voltage of 8–12 kV, energy density < 0.08 mJ/mm^2^, and pulse frequencies of 500, 200, or 120 pulses. Fasting blood glucose levels were monitored during the treatment period. To adhere to the 3R (Replacement, Reduction, Refinement) principle, each experimental group contained at least 3 rats at baseline, The specific group sizes for each parameter set were as follows: (1) Safety test in normoglycemic rats (Table 4): Normal control (*n* = 3), 8 kV/500 pulses (*n* = 3), 10 kV/500 pulses (*n* = 3), and 12 kV/500 pulses (*n* = 3). (2) Efficacy and safety in diabetic rats 500 pulses (Table 5): Normal control (*n* = 3), diabetic model (*n* = 3), 8 kV/500 pulses (*n* = 6), 10 kV/500 pulses (*n* = 3), and 12 kV/500 pulses (*n* = 3). The larger sample size for the 8 kV/500 pulses group was chosen because pilot tests indicated possible partial mortality, requiring more animals to obtain evaluable data. (3) Diabetic rats 200 pulses (Table 6): Normal control (*n* = 3), diabetic model (*n* = 3), 6 kV/200 pulses (*n* = 3), and 8 kV/200 pulses (*n* = 3). (4) Diabetic rats 120 pulses (Table 7): Normal control (*n* = 3), diabetic model (*n* = 3), 6 kV/120 pulses (*n* = 6), and 8 kV/120 pulses (*n* = 6). Larger group sizes were used here to better assess treatment effects after reducing the pulse number.

### 2.5. Establishment of the T2DM Model in OLETF Rats

The OLETF rat model was used for efficacy assessment because it represents a spontaneous, non-induced form of T2DM that more closely mimics the progressive metabolic deterioration seen in human patients, thereby providing higher translational relevance than acute chemically induced models. Male OLETF rats were divided into normal control and model groups. The model group was continuously fed a high-sucrose, high-fat diet (composition: 20% sucrose, 10% lard, 0.5% sodium cholate, 1% cholesterol) for 55 weeks until fasting blood glucose levels reached 6.6–8 mmol/L. Subsequently, a single low-dose intraperitoneal injection of streptozotocin (10 mg/kg) dissolved in citrate buffer was administered to further induce Type 2 diabetes. Before modeling, all rats were fasted for 12 h with free access to water. Rats in the normal control group received citrate buffer only, while all others received STZ in citrate buffer. Three days post-injection, blood glucose levels were measured via tail vein puncture using a glucometer. All model rats with sustained blood glucose levels > 11.1 mmol/L were included in the experiment. To ensure unbiased allocation, rats meeting the inclusion criteria were randomly assigned to groups using a random number table. The final groups were: normal control group (Normal, *n* = 3), T2DM model group without treatment (Model, *n* = 3), and shock wave treatment group (SW, *n* = 6). The Model and SW groups underwent the same high-fat diet and STZ injection protocol.

### 2.6. ELESW Treatment Protocol for OLETF Rats

Rats were anesthetized with 2.0% isoflurane inhalation, positioned in lateral recumbency on an animal board, and the abdominal hair over the liver and pancreas regions was shaved. After skin exposure, medical ultrasound coupling gel was applied. The shock wave transducer was positioned over the liver and pancreas areas using the anatomical landmarks (xiphoid process and left costal margin) as described in Section 2.3, ensuring consistent probe placement for each treatment session. The treatment parameters were: output voltage 7 kV; energy flux density < 0.08 mJ mm^2^; and a pulse regimen of 30 consecutive pulses followed by a 30-s interval, with 150 pulses constituting one treatment session. Each T2DM rat received 18 consecutive days of treatment, followed by a 7-day observation period with continued blood glucose monitoring. All treatments were performed by a single operator who was aware of group allocation. However, all outcome assessments—including fasting and postprandial blood glucose measurements, glucose tolerance test (GTT), insulin tolerance test (ITT), histopathological analysis, and qPCR analysis—were conducted by different investigators who were blinded to the treatment group identities.

### 2.7. Glucose Tolerance Test (GTT) and Insulin Tolerance Test (ITT)

To assess glucose clearance capacity before and after treatment, a GTT was performed after a 12-h fast. A 20% glucose solution was administered intraperitoneally (2 g/kg). Blood glucose levels were measured at 0, 15, 30, 60, and 120 min using a glucometer, and the area under the curve (AUC) was calculated to quantify overall glucose tolerance.

To evaluate peripheral tissue insulin sensitivity before and after treatment, an ITT was conducted after a 6-h fast. Insulin dissolved in physiological saline was administered intraperitoneally (0.5 U/kg). Blood glucose levels were measured at 0, 15, 30, 60, and 120 min, and the AUC was calculated to assess insulin sensitivity.

### 2.8. Serum Insulin Measurement and Insulin Resistance Index

Fasting serum insulin levels were measured using a commercial rat-specific sandwich ELISA kit. Absorbance at 450 nm was determined using a microplate reader, with the intensity being proportional to the insulin concentration in the sample.

The fasting serum insulin concentrations, together with the fasting blood glucose levels, were used to calculate the homeostatic model assessment of insulin resistance (HOMA-IR) index, a key metric for evaluating systemic insulin sensitivity [21]. The formula used was:

HOMA-IR = [Fasting blood glucose (mmol/L) × Fasting insulin (mU/L)]/22.5

### 2.9. Histopathological Sectioning

Fresh pancreatic, hepatic, and renal tissues were fixed in 4% paraformaldehyde for over 24 h. Following dehydration, tissues were embedded in paraffin and sectioned at a thickness of 4 μm. Sections were deparaffinized and stained with haematoxylin and eosin (H&E) according to the manufacturer’s protocol.

### 2.10. mRNA Detection in Pancreatic, Hepatic, and Renal Tissues of T2DM Rats

Total RNA was extracted from pancreatic, liver, and kidney tissues using a commercial kit according to the manufacturer’s instructions. cDNA was synthesized via reverse transcription. qPCR amplification was performed using the following protocol: initial denaturation at 95 °C for 2 min, followed by 40 cycles of denaturation at 95 °C for 15 s and annealing/extension at 60 °C for 30 s. The CT values were recorded, and the relative expression of target genes was calculated using the 2^−ΔΔCT^ method. The number of animals per group (*n*) is as described in Section 2.4 and Section 2.5. Three technical replicates were performed for each animal sample, and the mean of the technical replicates was used as one data point for statistical analysis. Primer sequences are listed in Table 1.

### 2.11. Effect of ELESW on HepG2 Cell Viability

HepG2 cells were cultured in DMEM medium supplemented with 10% fetal bovine serum (FBS) and 1% penicillin/streptomycin at 37 °C in a 5% CO_2_ incubator. Cells were passaged upon reaching 80% confluence.

Cells were seeded in 96-well plates at a density of 1 × 10^5^ cells per well and cultured for 24 h. After cell attachment, the supernatant was discarded and replaced with serum-free DMEM medium. Cells were then subjected to low-energy shock waves at different pulse frequencies (50, 100, 150, 200, 300, 400 pulses). Following treatment, cells were cultured for an additional 24 h. The supernatant was then removed, and 20 μL of MTT solution was added to each well, followed by incubation for 4 h. After removing the MTT solution, 150 μL of DMSO was added to each well. Plates were placed on a shaker and agitated at 37 °C for 10 min. Absorbance at 490 nm was measured for each well using a microplate reader.

### 2.12. Establishment of the IR-HepG2 Cell Model and Experimental Grouping

An insulin-resistant cell model was established based on previously reported methods [22,23] to determine suitable insulin concentration and exposure duration. HepG2 cells were seeded in 96-well plates at a density of 1 × 10^5^ cells per well and cultured for 24 h at 37 °C in a 5% CO_2_ incubator. Cells were then serum-starved for 12 h using serum-free medium. Except for the normal control and normal shock wave groups, all other groups were treated with DMEM medium containing 10^−6^ mol/L insulin and 33 mmol L^−1^ glucose for 24 h to induce insulin resistance. Subsequently, cells were subjected to shock wave treatment (300 pulses, 7 kV, energy flux density < 0.08 mJ/mm^2^) and cultured for another 24 h. The experimental groups were as follows: normal control group (Normal), normal shock wave group (Normal Shock Wave, NSW), model group (Model), and model shock wave group (Model Shock Wave, MSW). For cell experiments and qPCR analyses, three independent biological replicates were performed (*n* = 3). In each independent experiment, three technical replicates were included for each condition, and the mean of the technical replicates was used as one data point for statistical analysis. The same procedure was applied to all subsequent cell experiments and qPCR analyses.

### 2.13. Detection of Key Enzyme mRNA Related to Glucose Metabolism in IR-HepG2 Cells

HepG2 cells were seeded in 6-well plates at a density of 2 × 10^3^ cells per well and cultured in DMEM medium containing 10% FBS for 24 h. Grouping and treatments were performed as described in Section 3.4.2. After treatment, total RNA was extracted using a commercial kit according to the manufacturer’s instructions, and cDNA was synthesized via reverse transcription. qPCR amplification was performed under the following conditions: initial denaturation at 95 °C for 2 min, followed by 40 cycles of denaturation at 95 °C for 15 s and annealing/extension at 60 °C for 30 s. The CT values were recorded, and the relative expression of target genes was calculated using the 2^−ΔΔCT^ method. Primer sequences are listed in Table 2.

### 2.14. Establishment of the HG-HepG2 Cell Model and Experimental Grouping

A high-glucose-induced cell damage model was established by treating cells with 50 mmol/L glucose [24,25,26]. HepG2 cells were seeded in 96-well plates at a density of 1 × 10^5^ cells per well and cultured for 24 h at 37 °C in a 5% CO_2_ incubator. The supernatant was then discarded, and except for the normal control group, all other groups were treated with serum-free DMEM medium containing 50 mmol/L glucose. Cells were subsequently subjected to shock wave treatment (300 pulses, 7 kV, energy flux density < 0.08 mJ/mm^2^) and cultured for another 24 h. The experimental groups were as follows: normal control group (Normal), normal shock wave group (Normal Shock Wave, NSW), model group (Model; 50 mmol/L glucose), and model shock wave group (Model Shock Wave, MSW; 50 mmol/L glucose + shock wave).

### 2.15. Measurement of ALT, AST, and Glycogen Content

Alanine aminotransferase (ALT) and aspartate aminotransferase (AST) activities were measured using commercial assay kits following the manufacturer’s protocols. Enzyme activities were calculated from the colorimetric reactions.

Glycogen content was determined using the anthrone method according to the kit instructions. Glycogen was extracted with a strongly alkaline solution, and the content was measured under strongly acidic conditions using the anthrone chromogenic reagent.

### 2.16. Detection of Glucose Metabolism and Pyroptosis-Related mRNA in HG-HepG2 Cells

HepG2 cells were seeded in 6-well plates at a density of 2 × 10^3^ cells per well, cultured with medium for 24 h, and then grouped and treated according to the methods described in the relevant section. After treatment, total RNA was extracted using a commercial kit according to the manufacturer’s instructions, and cDNA was synthesized via reverse transcription. qPCR amplification was performed under the following conditions: initial denaturation at 95 °C for 2 min, followed by 40 cycles of denaturation at 95 °C for 15 s and annealing/extension at 60 °C for 30 s. The CT values were recorded, and the relative expression of target genes was calculated using the 2^−ΔΔCT^ method. Primer sequences are listed in Table 2 and Table 3.

### 2.17. Statistical Analysis

Data were analyzed using SPSS 24 software and are presented as mean ± s.e.m. Graphs were generated using GraphPad Prism 9. Prior to analysis, normality (Shapiro–Wilk test) and homogeneity of variances (Levene’s test) were checked. For comparisons among multiple independent groups, one-way ANOVA was performed, followed by LSD and Duncan’s multiple range tests for post hoc comparisons. For repeated-measures data (e.g., blood glucose over time), a repeated-measures ANOVA was used. When the sphericity assumption was violated, Greenhouse–Geisser correction was applied. *p* < 0.05 was considered statistically significant. All experiments were conducted under blinded conditions for outcome assessment, and group sizes adhered to the 3R principle as described in the respective sections.

## 3. Results

### 3.1. Parameter Optimization and Safety Evaluation of ELESW Therapy for T2DM Rats

To assess whether ELESW affects normal physiological functions, safety tests were first performed on SD rats with normal blood glucose levels. As shown in Table 4, after applying shock waves at voltages of 8 kV, 10 kV, and 12 kV with 500 pulses, blood glucose levels were monitored at different time points over 18 days. The results showed that there were no significant differences in blood glucose levels between the shock wave-treated groups (8 kV, 10 kV, and 12 kV) and the normal control group (*p* > 0.05). These findings indicate that low-energy shock waves (8–12 kV) do not affect normal physiological functions or blood glucose levels in normoglycemic rats.

**Table 4:** Effects of low, medium, and high voltage and 500 low-energy shock waves on blood glucose in normal rats.

Group/Day	Day 0	Day 3	Day 6	Day 13	Day 18
Control	5.43 ± 0.15	5.37 ± 0.21	5.20 ± 0.26	5.47 ± 0.25	5.37 ± 0.15
8 kV/500 pulses	5.20 ± 0.46	5.13 ± 0.47	5.07 ± 0.15	5.43 ± 0.35	5.13 ± 0.31
10 kV/500 pulses	5.17 ± 0.25	5.13 ± 0.38	5.27 ± 0.15	5.33 ± 0.49	5.43 ± 0.25
12 kV/500 pulses	5.10 ± 0.20	5.07 ± 0.35	5.00 ± 0.17	5.57 ± 0.23	5.43 ± 0.32

Note: Each group started with 3 rats (*n* = 3), and no mortality occurred. Data are presented as mean ± s.e.m. No significant differences were observed within any group compared with Day 0 (*p* > 0.05).

After confirming that ELESW (8–12 kV/500 pulses) had no effect on the physiological functions of normal rats, this study further evaluated their therapeutic efficacy in T2DM model rats. As shown in Table 5, shock wave treatment at 8 kV, 10 kV, and 12 kV with 500 pulses easily caused death in diabetic model rats when initial blood glucose was high. The 8 kV/500 pulses group started with a total of 6 rats. Among these, 3 rats had initial blood glucose > 20 mmol/L; 1 of these 3 died (33.3% mortality within this high-glucose subgroup). The other 3 rats had initial blood glucose < 20 mmol/L (12.60 ± 0.66 mmol/L); none died (0% mortality). The 10 kV/500 pulses and 12 kV/500 pulses groups each started with 3 rats, all of which had initial blood glucose > 20 mmol/L, and all died (100% mortality). Most of the deceased rats stopped eating within 3 days after treatment, and necropsy revealed diffuse hemorrhage in the intestinal mucosa as well as petechial hemorrhages in the pancreas, liver, and other parenchymal organs. These observations suggest that under diabetic conditions, high initial blood glucose levels may be associated with increased fragility of parenchymal organs to mechanical stimulation, thereby narrowing the safety window for shock wave application.

Intragroup comparisons with Day 0 showed that the normal group had no significant changes in blood glucose at any time point (*p* > 0.05), indicating that shock waves did not affect blood glucose in normal rats. In the model group, blood glucose gradually increased with disease progression and became significantly higher than Day 0 by Day 18 (*p* < 0.01), confirming the continuous deterioration of the diabetic model. In the 8 kV/500 pulses treatment group, rats with an initial blood glucose of approximately 24 mmol/L showed a decrease in blood glucose on Day 3 after shock wave treatment (but with a mortality rate of 33.3% and reduced sample size), followed by large fluctuations; no statistical comparisons were performed due to insufficient sample size. In contrast, rats with an initial blood glucose of approximately 12.6 mmol/L had blood glucose levels significantly lower than Day 0 starting from Day 6 (*p* < 0.05 on Day 6, *p* < 0.01 on Day 13 and Day 18). Consequently, subsequent experiments needed to strictly control the blood glucose range and adjust the shock wave energy.

**Table 5:** Effects of low, medium, and high voltage and 500 low-energy shock waves on blood glucose in T2DM rats.

Group/Day	Day 0	Day 3	Day 6	Day 13	Day 18
Control	4.97 ± 0.21	5.00 ± 0.26	4.97 ± 0.25	4.90 ± 0.10	5.07 ± 0.12
Model	25.77 ± 1.97	25.70 ± 1.32	27.43 ± 1.10	27.50 ± 0.80	29.60 ± 0.62 **
8 kV/500 pulses	24.00 ± 0.66	18.85 ± 2.05 (33.3% mortality)	25.25 ± 1.63	19.45 ± 0.35	27.30 ± 4.81
	12.60 ± 0.66	9.37 ± 2.06	9.00 ± 0.70 *	8.10 ± 0.80 **	6.90 ± 0.20 **
10 kV/500 pulses	20.43 ± 1.70	100% mortality			
12 kV/500 pulses	19.20 ± 2.05	100% mortality			

Note: Intra-group comparison versus Day 0: * *p* < 0.05, ** *p* < 0.01. (The same below). For the 8 kV/500 pulses (high glucose) subgroup, values at Day 3 and later are mean ± s.e.m. of the 2 surviving rats. No statistical comparison was performed for this subgroup due to small sample size (*n* = 2). The 8 kV/500 pulses (lower glucose) subgroup had *n* = 3 at all time points.

As shown in Table 6 and Table 7, when the shock wave parameters were significantly reduced to 6–8 kV with 200 or 120 pulses, the mortality rate of T2DM rats markedly decreased. Intra-group comparison analysis revealed that, in the 200-pulse experiment, the normal group showed no significant change in blood glucose (*p* > 0.05). In the model group, blood glucose on Day 14 was significantly higher than on Day 0 (*p* < 0.05). In the treatment groups (6 kV and 8 kV), blood glucose on Day 14 was significantly lower than on Day 0 (*p* < 0.05 for all), indicating that under safe parameters, ELESW may help improve blood glucose.

In the 120-pulse experiment, the normal group showed no changes at any time point. The model group exhibited continuously increasing blood glucose levels, which were significantly higher on Day 7 than on Day 0 (*p* < 0.05). For rats with blood glucose over 20 mmol/L, 6 kV/120 pulses did not reduce blood glucose (no significant difference compared to Day 0 at any time point; *p* > 0.05), while 8 kV/120 pulses caused a significant rise in blood glucose on Day 7 (*p* < 0.05), with this group experiencing a mortality rate of 33.3%. For rats with blood glucose around 13–14 mmol/L, 120 pulses at 6 kV and 8 kV all significantly decreased blood glucose: on both Day 2 and Day 7, blood glucose was significantly lower than on Day 0 (*p* < 0.05) in all three treatment groups.

**Table 6:** Effects of low voltage with 200 low-energy shock waves on blood glucose in T2DM rats.

Group/Day	Day 0	Day 14
Control	5.07 ± 0.21	5.00 ± 0.10
Model	12.83 ± 1.31	16.43 ± 0.78 *
6 kV/200 pulses	12.53 ± 4.17	8.87 ± 2.32 *
8 kV/200 pulses	11.17 ± 0.87	8.50 ± 1.37 *

Note: Each group started with *n* = 3, and no mortality occurred. Data are presented as mean ± s.e.m. * *p* < 0.05 vs. Day 0 (intragroup comparison).

**Table 7:** Effects of low voltage with 120 low-energy shock waves on blood glucose in T2DM rats.

Group/Day	Day 0	Day 2	Day 7
Control	5.03 ± 0.15	5.03 ± 0.21	4.83 ± 0.15
Model	23.20 ± 2.21	24.87 ± 1.80	26.90 ± 1.35 *
6 kV/120 pulses	26.33 ± 3.28	29.70 ± 3.34	28.23 ± 1.38
	13.87 ± 1.04	12.33 ± 0.86	10.53 ± 1.12 *
8 kV/120 pulses	24.00 ± 2.10	26.10 ± 2.26 (33.3% mortality)	28.75 ± 1.20 *
	12.60 ± 0.66	11.40 ± 0.53	9.47 ± 0.45 *

Note: Normal and model groups: *n* = 3 for each. For the 6 kV/120 pulses and 8 kV/120 pulses groups: *n* = 6 for each, with two subgroups (*n* = 3 each). One death occurred in the high-glucose subgroup of the 8 kV/120 pulses group (*n* = 2 survivors). No other mortality. Data are mean ± s.e.m. * *p* < 0.05 vs. Day 0.

In summary, the SD rat experiments demonstrated that the safe parameter range for ELESW was 6–8 kV with 120–200 pulses. It was effective in individuals with lower initial blood glucose levels but either ineffective or potentially harmful in those with extremely high blood glucose. The following ELESW parameters were selected: an output voltage of 7 kV and a pulse regimen of 30 stimuli followed by a 30-s interval (total pulses: 150). The electromagnetic shock equipment was equipped with a focusing lens and a dual-layer damping interface, yielding an energy flux density of <0.08 mJ/mm^2^.

### 3.2. Effects of ELESW on Hyperglycaemia and Organ Function in a Rat Model of Type 2 Diabetes

#### 3.2.1. Effect of ELESW on Fasting Blood Glucose in T2DM OLETF Rats

**Figure 1:**
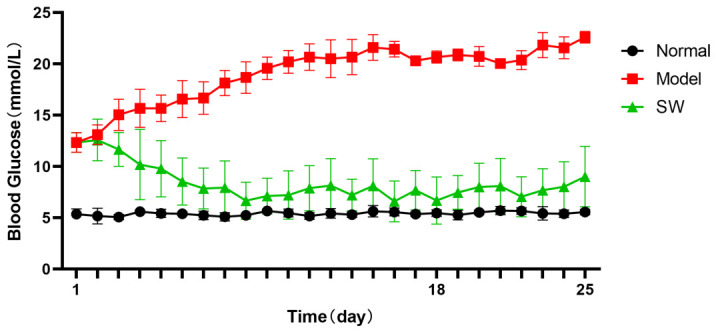
Effect of ELESW treatment on FBG in T2DM rats.

As shown in Figure 1, the fasting blood glucose levels in the model group were significantly increased (>11.1 mmol/L) at the start of the treatment compared with the control group, indicating that high-sugar and high-fat feeding combined with low-dose STZ injection successfully induced a T2DM model. Following one week of ELESW therapy, the fasting blood glucose level of the treatment group gradually declined and then stabilized. The fasting blood glucose level remained relatively stable during the one-week observation period after treatment was stopped on day 18. In contrast, the fasting blood glucose of the model group increased to over 20 mmol/L on day 10. These results demonstrated that ELESW treatment significantly improved fasting blood glucose levels in T2DM rats.

#### 3.2.2. Effect of ELESW on Postprandial Blood Glucose in T2DM Rats

**Figure 2:**
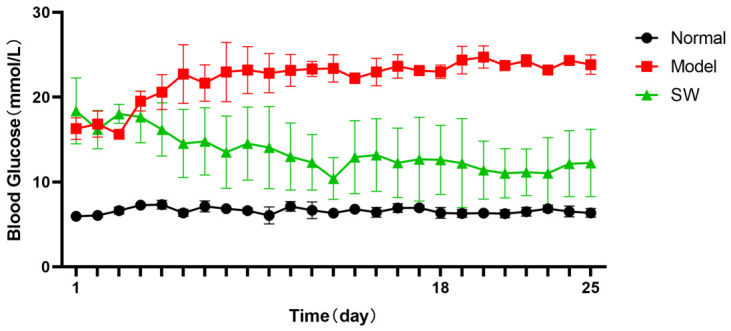
Effect of ELESW treatment on PBG in T2DM rats.

As shown in Figure 2, the blood glucose level in the model group increased sharply at 2 h postprandially and remained significantly higher than that in the control group. In contrast, the postprandial glucose of the treatment group gradually declined following ELESW therapy. After 10 days of treatment, postprandial glucose stabilized at near-baseline levels. During the one-week period after treatment cessation, the blood glucose level of the treatment group was slightly above that of the normal control group, but significantly lower than that of the model group. These findings demonstrate that ELESW therapy significantly ameliorated postprandial hyperglycaemia associated with T2DM.

#### 3.2.3. Effect of ELESW on Body Weight in T2DM Rats

**Figure 3:**
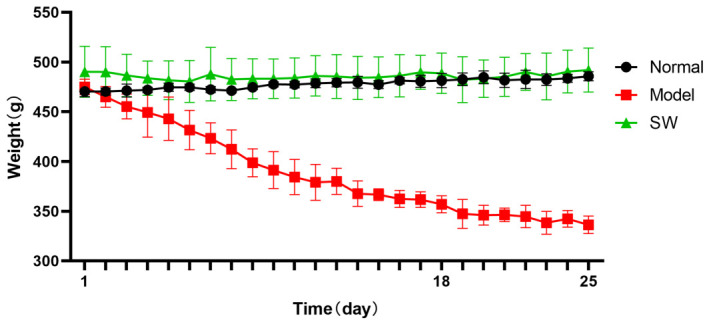
Effect of ELESW treatment on body weight in T2DM rats.

As shown in Figure 3, the body weight of the untreated model group was markedly reduced compared with the normal control group and showed progressive weight loss associated with sustained hyperglycaemia. In contrast, the body weight of the ELESW treatment group decreased only modestly during the first 5 days of therapy and then stabilized. These results suggest that ELESW therapy may alleviate weight loss, possibly because ELESW regulates blood glucose, since a high blood glucose environment may cause tissue damage and lead to continuous weight loss in T2DM rats.

#### 3.2.4. Effect of ELESW on Glucose Tolerance and Insulin Sensitivity in T2DM Rats

**Figure 4:**
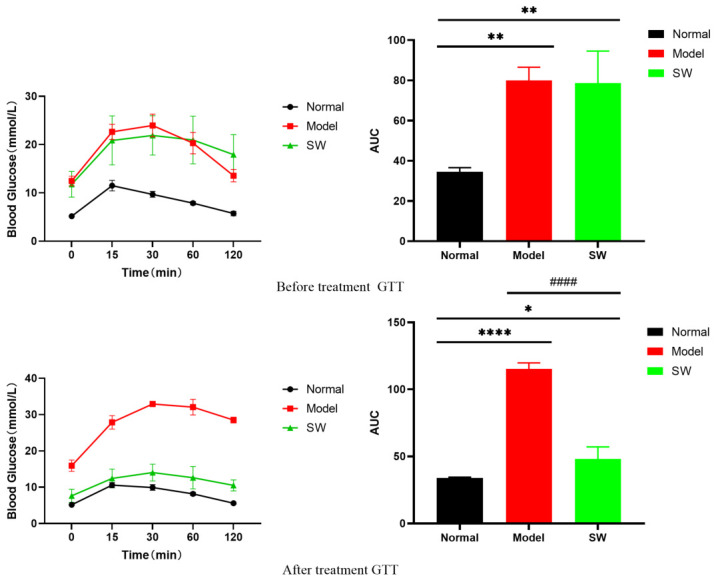
GTT in T2DM rats before and after ELESW therapy. Note: * *p* < 0.05, ** *p* < 0.01, **** *p* < 0.0001 vs. normal control group; #### *p* < 0.0001 vs. model group. The experimental groups were defined as follows: the “Normal” group received no treatment; the “Model” group was injected with STZ but received no subsequent therapy; and the “SW” (Shock Wave) group was injected with STZ and subsequently treated with shock waves.

As shown in Figure 4, the glucose tolerance test (GTT) before treatment revealed that both the Model and Shock Wave groups exhibited significantly higher blood glucose peaks compared to the Normal group, whereas the Normal group demonstrated the smallest area under the curve (AUC), indicating normal glucose processing capacity. The Model and Shock Wave groups showed significantly larger AUCs than the Normal group (*p* < 0.01), with no significant difference between the two diabetic groups (*p* > 0.05), demonstrating substantial glucose intolerance in T2DM rats. Following the intervention, the Shock Wave group exhibited a markedly reduced blood glucose peak, and its AUC was significantly smaller than that of the Model group (*p* < 0.0001). Although the AUC of the Shock Wave group remained larger than that of the Normal group, it approached normal levels (*p* < 0.05), indicating that ELESW treatment effectively improved glucose tolerance in T2DM rats.

**Figure 5:**
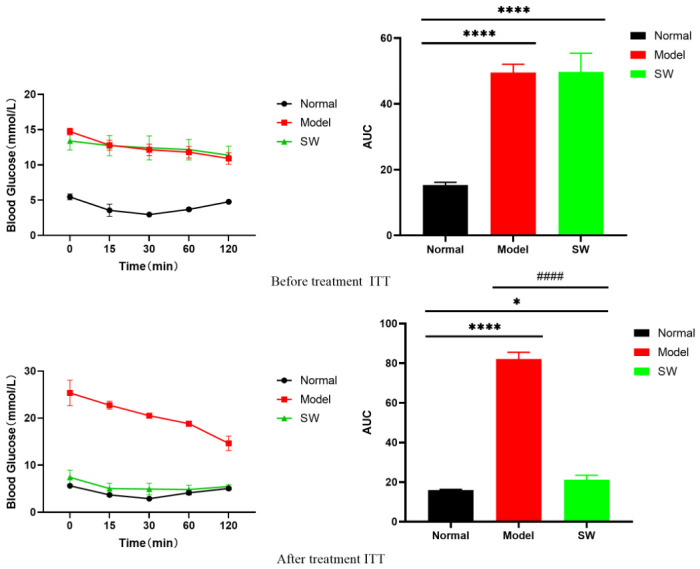
ITT in T2DM rats before and after ELESW therapy. Note: * *p* < 0.05, **** *p* < 0.0001 vs. normal control group; #### *p* < 0.0001 vs. model group.

As shown in Figure 5, the insulin tolerance test (ITT) before treatment demonstrated that both the Model and Shock Wave groups exhibited significantly reduced insulin sensitivity compared to the Normal group. The Normal group displayed the smallest AUC, indicating normal insulin sensitivity, while both the Model and Shock Wave groups showed significantly larger AUCs than the Normal group (*p* < 0.01), with no significant difference between the two diabetic groups (*p* > 0.05), which confirmed that insulin response was impaired in T2DM rats. Following the intervention, the Shock Wave group demonstrated markedly improved insulin sensitivity, with its AUC being significantly smaller than that of the Model group (*p* < 0.0001). Although the AUC of the Shock Wave group remained larger than that of the Normal group, it approached normal levels (*p* < 0.05), demonstrating that ELESW treatment effectively enhanced insulin sensitivity in T2DM rats.

#### 3.2.5. Effect of ELESW on Serum Insulin and Insulin Resistance in T2DM Rats

As shown in Table 8, compared with the Normal group, the Model group exhibited significantly elevated levels of FBG, fasting serum insulin (FINS), and homeostatic model assessment of insulin resistance (HOMA-IR) (*p* < 0.01). Although the HOMA-IR in the Shock Wave group remained significantly higher than that in the Normal group (*p* < 0.05), it approached normal levels, while no significant differences were observed in the other parameters (*p* > 0.05). In comparison with the Model group, the Shock Wave group demonstrated significant reductions in fasting blood glucose, fasting serum insulin, and HOMA-IR (*p* < 0.01), indicating that the treatment markedly improved insulin resistance in T2DM rats.

**Table 8:** Effect of ELESW treatment on FBG, fasting FINS, and insulin resistance index in T2DM rats.

Group	Normal	Model	SW
FBG (mmol/L)	5.57 ± 0.25 ^B^	22.6 ± 0.56 ^A^	9.02 ± 2.94 ^B^
FINS (mU/L)	18.02 ± 0.91 ^B^	25.94 ± 0.77 ^A^	19.58 ± 2.71 ^B^
HOMA-IR	4.45 ± 0.12 ^Ba^	26.06 ± 0.93 ^A^	7.55 ± 1.49 ^Bb^

Note: Different uppercase letters (e.g., A vs. B) indicate a statistically highly significant difference between groups (*p* < 0.01). Different lowercase letters (e.g., a vs. b) indicate a statistically significant difference (*p* < 0.05). Groups sharing the same uppercase letter (e.g., B and B) are not significantly different (*p* > 0.05). *n* = 3 for Normal and Model groups, *n* = 6 for SW group. Data are presented as mean ± s.e.m.

#### 3.2.6. Effect of ELESW on Pancreatic, Hepatic, and Renal Histomorphology in Diabetic Rats

The pancreas regulates blood glucose by secreting insulin and glucagon in response to circulating glucose levels. Insulin, the body’s sole glucose-lowering hormone, is produced by β-cells, which constitute 60–80% of the pancreatic islet endocrine mass [27]. The liver serves as the central organ for glucose metabolism, coordinating glycogen synthesis and breakdown along with gluconeogenesis to maintain systemic glucose homeostasis [28]. The kidneys contribute to glucose regulation through gluconeogenesis and glucose reabsorption, though chronic hyperglycaemia can induce glomerular microvascular pathology leading to diabetic nephropathy [29].

**Figure 6:**
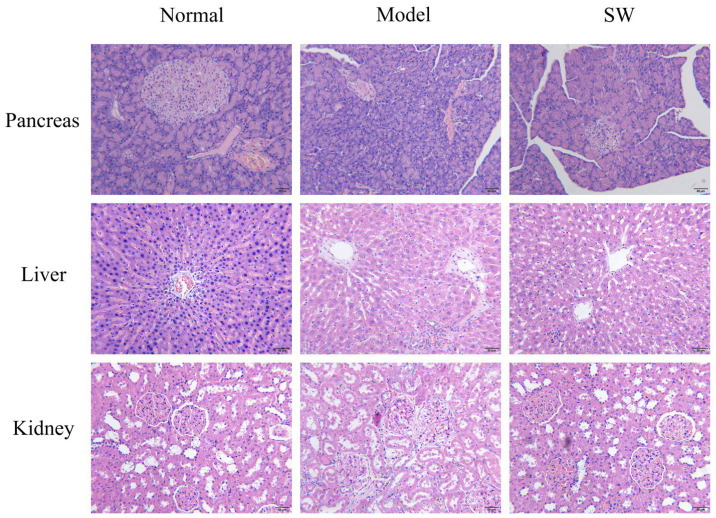
Representative histopathological sections of pancreas, liver, and kidney from each experimental group (H.E × 200, Bar = 50 μm).

Representative H&E staining images (Figure 6) showed that, compared with the normal control group, the model group exhibited morphological changes including smaller islet areas with apparent inflammatory infiltration, extensive hepatic inflammatory cell infiltration with lipid vacuolation, and renal corpuscle lobulation with tubular glycogen deposition. In the ELESW treatment group, the representative images suggested that islet areas appeared relatively larger, hepatic inflammatory infiltration seemed less pronounced, and renal morphology appeared improved compared with the model group. These histological observations are qualitative (based on representative images from each group) and were not subjected to blinded quantitative scoring. They suggest that ELESW treatment is associated with reduced diabetes-induced morphological damage to pancreatic, hepatic, and renal tissues, with larger islet areas and less inflammatory infiltration as observed in the representative sections.

#### 3.2.7. Effect of ELESW on the Expression of mRNA Factors of Pancreas Related to Insulin Synthesis and Secretion in T2DM Rats

Altered expression of insulin 1 (*INS1*), insulin 2 (*INS2*), glucose transporter 2 (*GLUT2*), and pancreatic and duodenal homeobox 1 (*PDX1*) following pancreatic injury is closely associated with β-cell function, insulin secretion, and glucose metabolism [30].

**Figure 7:**
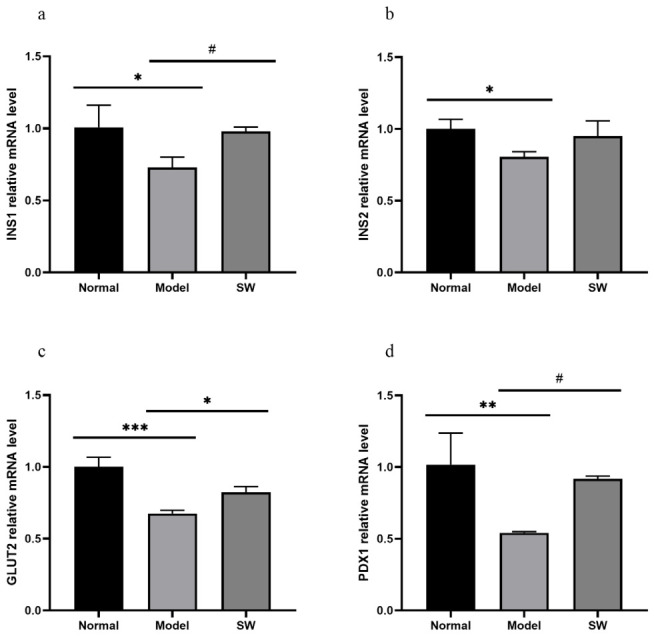
Effect of ELESW treatment on pancreatic mRNA expression in T2DM rats. (**a**) Relative mRNA expression level of *INS1*; (**b**) Relative mRNA expression level of *INS2*; (**c**) Relative mRNA expression level of *GLUT4*; (**d**) Relative mRNA expression level of *PDX1*. Note: * *p* < 0.05, ** *p* < 0.01, *** *p* < 0.001 vs. normal control group; # *p* < 0.05 vs. model group.

As shown in Figure 7a–d, compared with the Normal group, the Model group exhibited significantly reduced expression of *INS1* and *INS2* mRNA (*p* < 0.05), along with highly significant decreases in *PDX1* and *GLUT2* mRNA expression (*p* < 0.01 and *p* < 0.001, respectively). When compared with the Model group, the Shock Wave group demonstrated significantly improved expression of all measured markers except *INS2* (which showed no significant difference). These mRNA expression changes suggest that ELESW treatment may be associated with improved pancreatic β-cell function and islet recovery at the transcriptional level, which may contribute to glucose metabolism; however, direct functional confirmation is needed.

#### 3.2.8. Effect of ELESW on the Expression of mRNA Factors of Liver Related to Glycogen Synthesis and Gluconeogenesis in T2DM Rats

To evaluate the regulatory effect of ELESW on hepatic glucose metabolism, we analyzed key metabolic genes in the insulin signaling pathway, including insulin receptor (*INSR*), glucose transporter 4 (*GLUT4*), glycogen synthase kinase-3β (*GSK-3β*), glucokinase (*GK*), phosphoenolpyruvate carboxykinase (*PEPCK*), and peroxisome proliferator-activated receptor gamma coactivator 1-α (*PGC-1α*). Under insulin-resistant conditions, the expression or activity of these genes is typically dysregulated [31].

**Figure 8:**
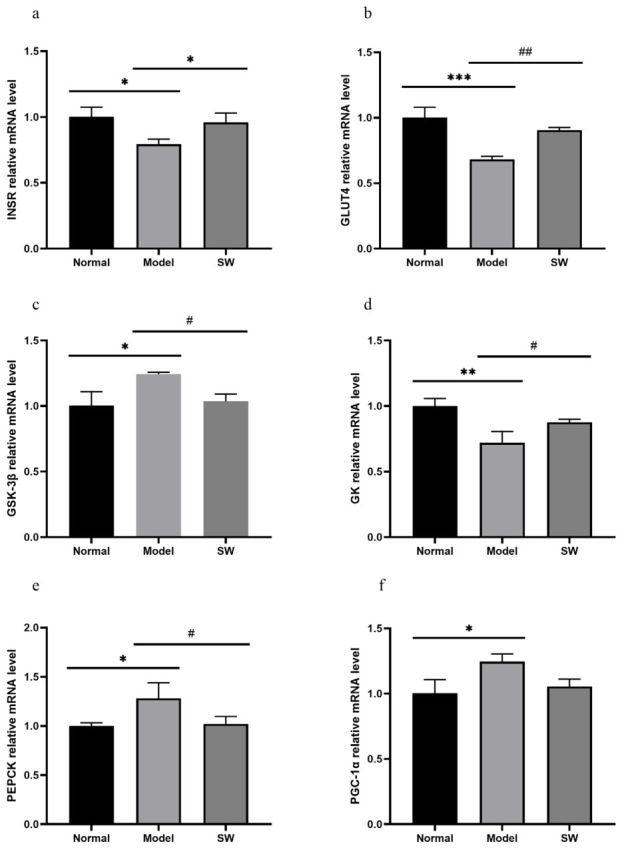
Effect of ELESW treatment on hepatic mRNA expression in T2DM rats. (**a**) Relative mRNA expression level of *INSR*; (**b**) Relative mRNA expression level of *GLUT4*; (**c**) Relative mRNA expression level of *GSK-3β*; (**d**) Relative mRNA expression level of *GK*; (**e**) Relative mRNA expression level of *PEPCK*; (**f**) Relative mRNA expression level of *PGC-1α*. Note: * *p* < 0.05, ** *p* < 0.01, *** *p* < 0.001 vs. normal control group; # *p* < 0.05, ## *p* < 0.01 vs. model group.

As shown in Figure 8a–f, compared with the Normal group, the Model group exhibited significantly decreased expression of *INSR* and *GSK-3β* mRNA (*p* < 0.05), along with highly significant reductions in *GLUT4* and *GK* mRNA (*p* < 0.001 and *p* < 0.01, respectively), while demonstrating significantly increased *PEPCK* mRNA (*p* < 0.05) and highly significantly elevated *PGC-1α* mRNA expression (*p* < 0.01). When compared with the Model group, the Shock Wave group showed significant improvements in all measured parameters except *PGC-1α* mRNA, which displayed no significant difference. These mRNA changes suggest that ELESW treatment may enhance insulin signaling, glucose transport, and glycogen synthesis, while suppressing gluconeogenic gene expression at the transcriptional level, which may be associated with amelioration of disordered glucose metabolism.

#### 3.2.9. Effect of ELESW on Expression of mRNA Factors Related to Renal Tubular Injury, Inflammatory Infiltration, and Fibrosis Markers in T2DM Rats

Diabetic nephropathy is one of the most common microvascular complications of diabetes. Chronic hyperglycaemia causes renal oxidative stress, inflammatory responses, and glomerular hyperfiltration [32,33], resulting in increased expression of injury and inflammation markers such as kidney injury molecule-1 (*KIM-1*), intercellular adhesion molecule-1 (*ICAM-1*), and transforming growth factor-β1 (*TGF-β1*).

**Figure 9:**
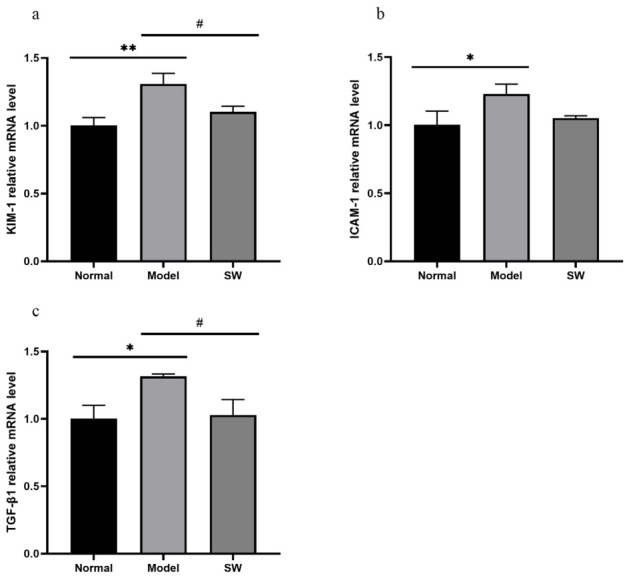
Effect of ELESW treatment on renal mRNA expression in T2DM rats. (**a**) Relative mRNA expression level of *KIM-1*; (**b**) Relative mRNA expression level of *ICAM-1*; (**c**) Relative mRNA expression level of *TGF-β1*. Note: * *p* < 0.05, ** *p* < 0.01 vs. normal control group; # *p* < 0.05 vs. model group.

As shown in Figure 9a–c, compared with the Normal group, the Model group exhibited significantly elevated expression of *ICAM-1* and *TGF-β1* mRNA (*p* < 0.05), along with a highly significant increase in *KIM-1* mRNA expression (*p* < 0.01). When compared with the Model group, the Shock Wave group demonstrated significantly reduced expression across all measured markers except *ICAM-1*, which showed no significant difference. These mRNA findings suggest that ELESW treatment may reduce inflammatory responses and could be associated with alleviation of hyperglycemia-induced renal damage; however, protein-level validation is required.

### 3.3. ELESW Ameliorated Hepatic Insulin Resistance by Altering mRNA Expression of Genes Related to Glucose Metabolism and Pyroptosis in HepG2 Cells

#### 3.3.1. Effect of ELESW on HepG2 Cell Viability and the Expression of mRNA Factors Related to Glucose Metabolism in IR-HepG2 Cells

**Figure 10:**
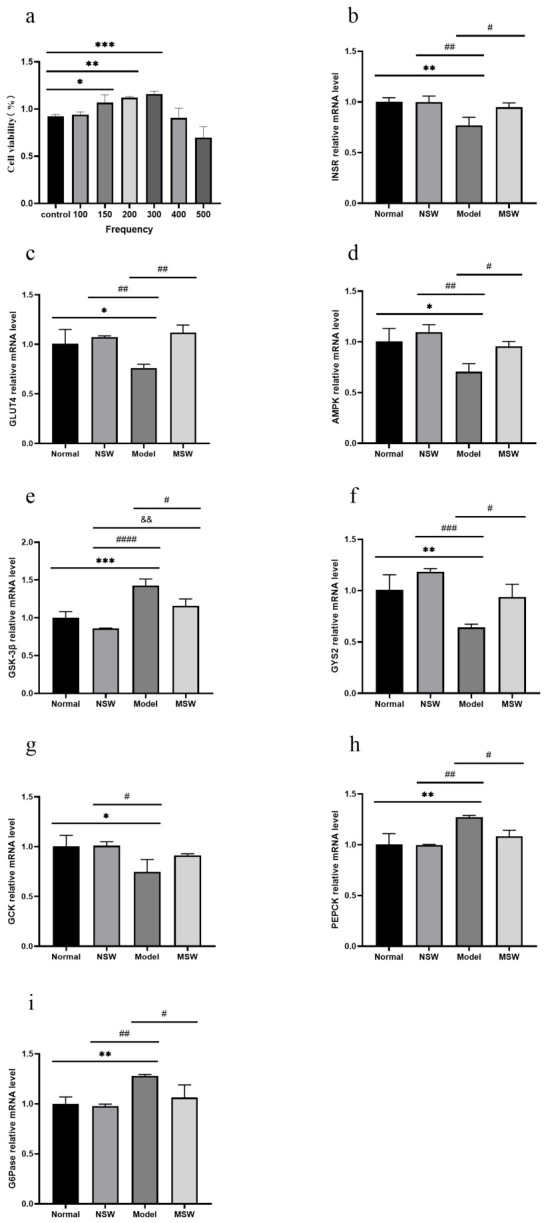
Effect of ELESW on HepG2 cell viability and glucose metabolism-related mRNA expression in IR-HepG2 cells. (**a**) Effect of ELESW with Different Pulse Frequencies on HepG2 Cell Viability; (**b**) Relative mRNA expression level of *INSR*; (**c**) Relative mRNA expression level of *GLUT4*; (**d**) Relative mRNA expression level of *AMPK*; (**e**) Relative mRNA expression level of *GSK-3β*; (**f**) Relative mRNA expression level of *GYS2*; (**g**) Relative mRNA expression level of *GCK*; (**h**) Relative mRNA expression level of *PEPCK*; (**i**) Relative mRNA expression level of *G6Pase*. Note: * *p* < 0.05, ** *p* < 0.01, *** *p* < 0.001 vs. normal control group; # *p* < 0.05, ## *p* < 0.01, ### *p* < 0.001, #### *p* < 0.0001 vs. model group; && *p* < 0.01 vs. normal shock wave group. The experimental groups were defined as follows: the “Normal” group received no treatment; the “NSW” (Normal Shock Wave) group was subjected to shock wave intervention without STZ injection; the “Model” group received an STZ injection without any subsequent therapy; and the “MSW” (Model Shock Wave) group was administered STZ and subsequently treated with shock waves.

As shown in Figure 10a, MTT assays following treatment with different pulse frequencies demonstrated that 150 (*p* < 0.05), 200 (*p* < 0.01), and 300 (*p* < 0.001) pulses significantly promoted HepG2 cell proliferation compared to the control group. Consequently, 300 pulses were selected for subsequent experiments.

To investigate the effect of ELESW on glucose metabolism in insulin-resistant (IR) HepG2 cells, we measured the mRNA expression levels of key genes involved in insulin signaling and glucose homeostasis using qPCR. These included *INSR*, *GLUT4*, AMP-activated protein kinase (*AMPK*), glycogen synthase 2 (*GYS2*), glucokinase (*GCK*), *PEPCK*, glucose-6-phosphatase (*G6Pase*), and *GSK-3β*.

As shown in Figure 10b–i, qPCR analysis revealed that compared with the Normal group, the Model group exhibited significantly downregulated expression of *GLUT4*, *AMPK*, and *GCK* mRNA (*p* < 0.05), along with highly significantly decreased levels of *INSR* and *GYS2* mRNA (*p* < 0.01), while demonstrating significantly upregulated *GSK-3β* mRNA expression (*p* < 0.001) together with highly significantly elevated *PEPCK* and *G6Pase* mRNA levels (*p* < 0.01). When compared with the Model group, the Shock Wave group showed significantly increased expression of *INSR*, *AMPK*, and *GYS2* mRNA (*p* < 0.05), along with highly significantly elevated *GLUT4* mRNA levels (*p* < 0.01), while displaying significantly reduced expression of *GSK-3β, PEPCK*, and *G6Pase* mRNA (*p* < 0.05). These changes in mRNA expression suggest that ELESW treatment may promote glucose uptake and transport, enhance glycogen synthesis, and suppress gluconeogenesis at the transcriptional level, indicating a potential amelioration of insulin resistance-associated metabolic disturbances.

#### 3.3.2. Effect of ELESW on the Expression of Proliferation, Pyroptosis, and Apoptosis-Related mRNA Factors in IR-HepG2 Cells

To investigate the effect of ELESW on cell fate decisions in insulin-resistant HepG2 cells, we assessed the mRNA expression levels of key markers associated with cell proliferation, pyroptosis, and apoptosis, including proliferating cell nuclear antigen (*PCNA*), cysteinyl aspartate-specific proteinase-1 (*caspase-1*), and cysteinyl aspartate-specific proteinase-3 (*caspase-3*).

**Figure 11:**
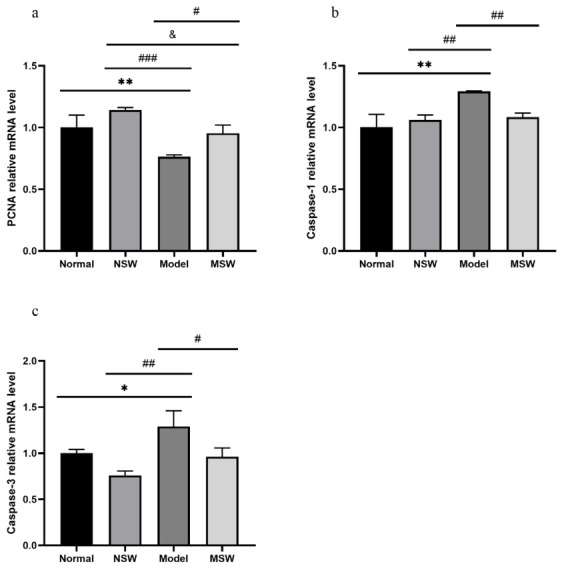
Effect of ELESW on mRNA expression of proliferation, pyroptosis, and apoptosis in IR-HepG2 cells. (**a**) Relative mRNA expression level of *PCNA*; (**b**) Relative mRNA expression level of *caspase-1*; (**c**) Relative mRNA expression level of *caspase-3*. Note: * *p* < 0.05, ** *p* < 0.01 vs. normal control group; # *p* < 0.05, ## *p* < 0.01, ### *p* < 0.001 vs. model group; & *p* < 0.05 vs. normal shock wave group.

As shown in Figure 11a–c, qPCR analysis revealed that compared with the Normal group, the Model group exhibited a highly significant decrease in *PCNA* mRNA expression (*p* < 0.01), along with significantly elevated levels of *caspase-1* mRNA (*p* < 0.01) and *caspase-3* mRNA (*p* < 0.05). When compared with the Model group, the Shock Wave group showed significantly increased *PCNA* mRNA expression (*p* < 0.05) together with significantly reduced mRNA levels of both *caspase-1* and *caspase-3* (*p* < 0.05). These mRNA expression changes collectively suggest that ELESW treatment may promote cellular proliferation and suppress pyroptosis- and apoptosis-related gene expression.

### 3.4. ELESW Was Associated with Alleviation of High-Glucose-Induced Hepatocyte Damage and Altered Metabolism- and Pyroptosis-Related Gene Expression in HepG2 Cells

#### 3.4.1. Effect of ELESW on ALT, AST, and Glycogen Content in HG-HepG2 Cells

Diabetes can induce liver injury. Sustained hyperglycaemia promotes oxidative stress and inflammatory responses in hepatocytes, leading to increased membrane permeability and elevated levels of alanine aminotransferase (ALT) and aspartate aminotransferase (AST). These inflammatory processes further exacerbate glucose metabolic disorders and impair hepatic glycogen synthesis [34,35,36].

**Figure 12:**
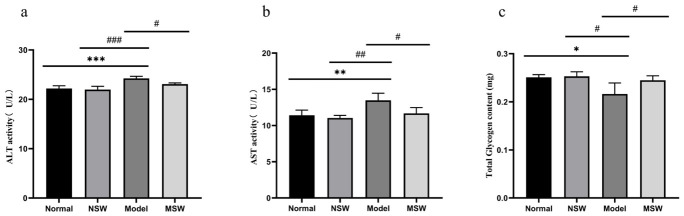
Effect of ELESW treatment on ALT, AST, and glycogen content in HG-HepG2 cells. (**a**) Effect of ELESW on ALT Activity in HG-HepG2 Cells; (**b**) Effect of ELESW on AST Activity in HG-HepG2 Cells; (**c**) Effect of ELESW on Total Glycogen Content in HG-HepG2 Cells. Note: * *p* < 0.05, ** *p* < 0.01, *** *p* < 0.001 vs. normal control group; # *p* < 0.05, ## *p* < 0.01, ### *p* < 0.001 vs. model group.

As shown in Figure 12a–c, compared with the Control group, the Model group exhibited significantly elevated levels of ALT and total glycogen content (*p* < 0.05), along with a highly significant increase in AST level (*p* < 0.01). When compared with the Model group, the Shock Wave group showed significantly reduced levels of both ALT and AST (*p* < 0.05), together with significantly increased total glycogen content (*p* < 0.05). These results indicate that ELESW treatment attenuates high-glucose-induced hepatocyte damage and is associated with increased glucose uptake and glycogen synthesis.

#### 3.4.2. Effect of ELESW on the Expression of mRNA Factors Related to Glucose Metabolism in HG-HepG2 Cells

To investigate the molecular mechanism by which ELESW ameliorated high-glucose-induced disturbances in HepG2 cell glucose metabolism, we examined the expression of key genes involved in glucose metabolism, including *INSR* and *GLUT4*.

**Figure 13:**
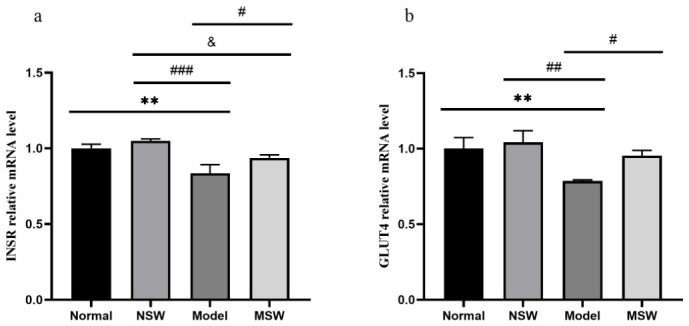
Effect of ELESW treatment on glucose metabolism-related mRNA expression in HG-HepG2 cells. (**a**) Relative mRNA expression level of *INSR*; (**b**) Relative mRNA expression level of *GLUT4*. Note: ** *p* < 0.01 vs. normal control group; # *p* < 0.05, ## *p* < 0.01, ### *p* < 0.001 vs. model group; & *p* < 0.05 vs. normal shock wave group.

As shown in Figure 13a,b, qPCR analysis revealed that compared with the Normal group, the Model group exhibited highly significant reductions in both *INSR* and *GLUT4* mRNA expression (*p* < 0.01). When compared with the Model group, the Shock Wave group showed significantly elevated expression levels of both *INSR* and *GLUT4* mRNA (*p* < 0.05). These mRNA results suggest that ELESW may enhance glucose transport capability, possibly through upregulation of *INSR* and *GLUT4* gene expression.

#### 3.4.3. Effect of ELESW on Expression of mRNA Factors Related to Pyroptosis, Apoptosis, and Proliferation in HG-HepG2 Cells

To investigate the molecular mechanism by which ELESW ameliorated high-glucose-induced damage in HepG2 cells, the expression of mRNA factors associated with pyroptosis, apoptosis, and proliferation was analyzed, including NLR family pyrin domain-containing 3 (*NLRP3*), *caspase-1*, gasdermin D (*GSDMD*), *caspase-3*, and *PCNA*.

**Figure 14:**
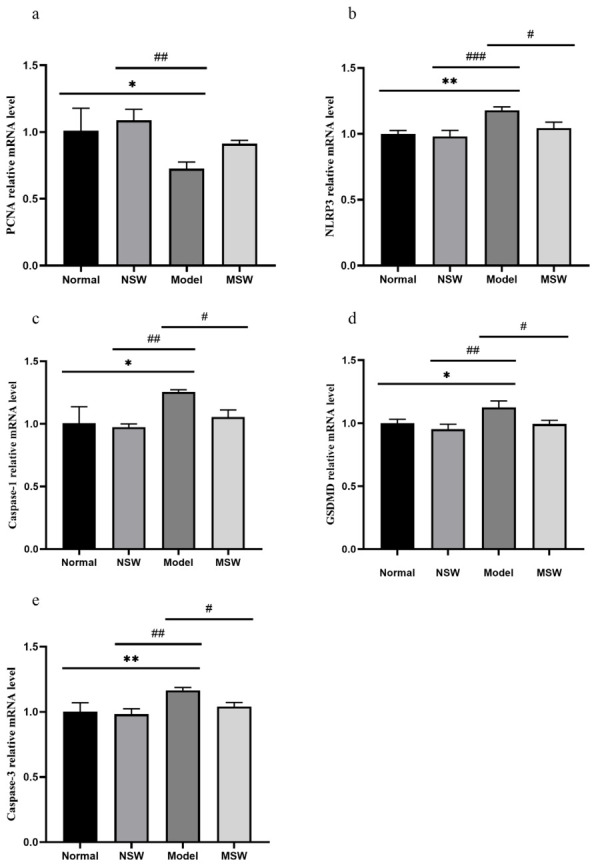
Effect of ELESW treatment on mRNA expression of proliferation, pyroptosis, and apoptosis in HG-HepG2 cells. (**a**) Relative mRNA expression level of *PCNA*; (**b**) Relative mRNA expression level of *NLRP3*; (**c**) Relative mRNA expression level of *caspase-1*; (**d**) Relative mRNA expression level of *GSDMD*; (**e**) Relative mRNA expression level of *caspase-3*. Note: * *p* < 0.05, ** *p* < 0.01 vs. normal control group; # *p* < 0.05, ## *p* < 0.01, ### *p* < 0.001 vs. model group.

As shown in Figure 14a–e, qPCR analysis revealed that compared with the Normal group, the Model group exhibited a significant decrease in *PCNA* mRNA expression (*p* < 0.05), along with highly significant increases in *NLRP3* and *caspase-3* mRNA levels (*p* < 0.01), together with significantly elevated expression of *caspase-1* and *GSDMD* mRNA (*p* < 0.05). When compared with the Model group, the Shock Wave group demonstrated significantly reduced mRNA expressions of *NLRP3*, *caspase-1*, *GSDMD*, and *caspase-3* (*p* < 0.05). These mRNA findings suggest that ELESW treatment may mitigate high-glucose-induced damage in HepG2 cells, potentially through downregulation of genes involved in pyroptosis and apoptosis.

## 4. Discussion

This study systematically investigated ELESW therapy as a non-invasive physical intervention for T2DM, using complementary animal models (SD and OLETF rats) and cellular models (IR-HepG2 and HG-HepG2). Our results showed that, under precisely optimized energy parameters, ELESW was associated with lower blood glucose levels and improved insulin resistance and glucose metabolic disorders in T2DM. The potential mechanisms, as suggested by mRNA and histopathological data, may involve promoting glucose utilization, enhancing insulin signal transduction, suppressing hepatic gluconeogenesis, attenuating local inflammation in the pancreas, liver, and kidneys, as well as protecting and promoting the proliferation and repair of pancreatic β-cells. Notably, ELESW treatment caused no detectable damage to normal tissues but specifically rectified metabolic disturbances under diabetic conditions and provided cytoprotection in hepatocytes under high-glucose stress.

A key finding of this study was that the safety and efficacy of ELESW therapy critically depended on precise energy control. ELESW levels exceeding the safety threshold damaged the fragile vasculature of T2DM rats—but not normoglycemic rats—leading to multi-organ hemorrhage. This observation suggests pathological alterations in vascular sensitivity to mechanical stress under diabetic conditions, consistent with previous reports that diabetes induces structural and functional abnormalities in vessel walls, thereby reducing their tolerance to external mechanical stress [37,38]. Compared with focused ultrasound stimulation, which targets specific neural plexuses [10,11], ELESW appears to exert broader multi-organ effects, but its narrower therapeutic window necessitates stricter parameter optimization. Although the high mortality (up to 100%) observed during parameter optimization in SD rats raises important safety and ethical concerns, but no mortality occurred in normoglycemic rats under the same stimulation parameters, indicating that diabetes-induced vascular fragility—rather than the shock wave itself—is the primary cause of hemorrhage and death. This finding has two implications. First, it establishes a narrow therapeutic window for ELESW in T2DM, which must be strictly adhered to in future studies. Second, it highlights the necessity of including safety-optimization phases in preclinical studies of mechanical therapies for diabetes. From an ethical standpoint, the severe outcomes were anticipated and approved by the ethics committee, with predefined humane endpoints and immediate euthanasia to minimize suffering. The 3R principle was followed by using the minimal number of animals required to define lethal thresholds (*n* = 3 per group, with *n* = 6 only for intermediate parameters). While the mortality itself is regrettable, the data obtained were essential to prevent the use of harmful parameters in subsequent efficacy studies. Future investigations should further refine protocols to reduce animal exposure to potentially lethal conditions, possibly by using real-time monitoring or lower-energy starting points.

After establishing safe parameters, ELESW treatment in OLETF rats significantly improved systemic glucose homeostasis, as evidenced by reduced fasting and postprandial blood glucose, enhanced insulin sensitivity, and attenuated pathological weight loss. These findings align with previous studies showing that non-invasive mechanical stimuli, including shock waves and ultrasound, facilitate pancreatic β-cell repair and improve insulin resistance in T2DM models [9,10,11,20]. Unlike pharmacological agents such as metformin, which primarily act on hepatic *AMPK*, ELESW appeared to exert simultaneous effects on the pancreas, liver, and kidneys, suggesting a distinct multi-target profile.

In the IR-HepG2 model, ELESW therapy was associated with amelioration of disordered glucose metabolism, potentially through a dual mechanism at the transcriptional level. First, it upregulated the mRNA expression of *INSR*, which may in turn influence the PI3K/AKT signaling axis and downstream enzymes involved in glucose uptake and utilization. This suggests a possible improvement in insulin signal transduction, a core strategy for reversing insulin resistance [39]. Second, ELESW increased the mRNA levels of *AMPK*, while concurrently promoting glycogen synthesis and suppressing the expression of key gluconeogenic enzymes (*PEPCK* and *G6Pase*), thus reducing hepatic glucose output [40,41]. These changes at the transcriptional level suggest that ELESW may affect AMPK-related pathways, similar to the action of metformin [42].

More importantly, our study revealed the tissue-protective and reparative functions of ELESW. Histopathological and qPCR analyses showed that ELESW significantly mitigated inflammatory cell infiltration and tissue damage in the pancreas, liver, and kidneys. Chronic low-grade inflammation is a known driver of both insulin resistance and β-cell dysfunction [43]. The observed increase in islet area, accompanied by reduced inflammation, suggests that ELESW may create a microenvironment conducive to β-cell survival and regeneration. This anti-inflammatory effect is consistent with previous reports that ELESW exerts anti-inflammatory, pro-angiogenic, and stem cell-activating effects in orthopedic and wound healing models [44,45,46], as well as its capacity to reduce pancreatic inflammation and apoptosis in diabetic models [20].

In high-glucose-stressed HepG2 cells, ELESW not only was associated with improved metabolic function through upregulation of *INSR* and *GLUT4* mRNA expression, but also may affect pyroptosis and apoptosis-related pathways by suppressing the mRNA levels of *NLRP3*, *caspase-1*, *GSDMD*, and *caspase-3*. These transcriptional changes, including the downregulation of *NLRP3*, *caspase-1*, *GSDMD*, and *caspase-3* mRNA, suggest that ELESW may affect the pyroptosis and apoptosis pathways at the transcriptional level.

However, mRNA changes do not directly reflect protein activation or cleavage events. For example, activation of the pyroptosis pathway requires cleavage of caspase-1 and gasdermin D (GSDMD-N), which cannot be inferred from mRNA data alone. Studies have shown that diabetes can induce hepatocyte pyroptosis by promoting oxidative stress-mediated NLRP3 inflammasome activation. Furthermore, hyperglycemia exacerbates acute liver injury by enhancing NLRP3 inflammasome activation in hepatic macrophages [47]. Previous research has shown that when endothelial cells are stimulated with shock waves at an energy flux density of 0.04–0.13 mJ/mm^2^, this not only enhances the expression of certain angiogenic factors but also reduces the expression of pro-apoptotic cytokines [48]. Furthermore, studies indicate that low-energy shock waves can induce intracellular actin cytoskeleton reorganization and Ca^2+^ influx through mechanical stimulation [49]. As a crucial second messenger, Ca^2+^ regulates multiple cellular processes, including proliferation, death, and energy metabolism [50,51,52]. It has also been reported that the mechanosensitive ion channel PIEZO1 serves as a primary receptor for hyperosmotic mechanical stress in corneal epithelial cells, and inhibition of PIEZO1 significantly reduces NLRP3 inflammasome-associated pyroptosis [53]. Based on these reports, it is tempting to hypothesize that ELESW might protect cells and improve tissue function through mechano-ion channel activation and Ca^2+^-dependent signaling, potentially counteracting T2DM-induced hyperglycemic damage. However, the present study did not experimentally test the involvement of PIEZO1, Ca^2+^ influx, or any specific mechanosensitive channels. Therefore, these proposed mechanisms remain speculative and should be considered as hypotheses to be tested in future studies, rather than as conclusions drawn from the current data.

Taken together, this study demonstrates that ELESW therapy, under optimized safe parameters, effectively lowers blood glucose and ameliorates insulin resistance in T2DM rats. At the transcriptional level, ELESW upregulates key genes involved in insulin signaling (e.g., *INSR*, *GLUT4*) and glucose metabolism (e.g., *AMPK*, *GYS2*), while downregulating genes associated with gluconeogenesis (e.g., *PEPCK*, *G6Pase*) and cell death pathways (e.g., *NLRP3*, *caspase-1*, *GSDMD*, *caspase-3*). These mRNA changes suggest that ELESW may exert its beneficial effects through modulation of the PI3K/AKT and AMPK signaling axes, as well as suppression of pyroptosis and apoptosis at the transcriptional level. Moreover, ELESW promotes islet repair and reduces inflammation in the pancreas, liver, and kidneys, suggesting a multi-organ protective effect. Nevertheless, this study has several limitations. First, the mechanistic conclusions are based exclusively on mRNA expression data; lack of protein-level validation precludes definitive statements about pathway activation. Second, the high mortality observed during parameter optimization raises ethical and safety concerns that were addressed by establishing a narrow therapeutic window. Third, no sham control group was included. Thus, non-specific effects of the procedures cannot be fully excluded. Nevertheless, the consistency of findings across different models supports the main conclusions. Future studies with a sham control are needed. Subsequent research should involve larger sample sizes, incorporate protein-level assays, and directly test the mechanotransduction hypotheses to facilitate clinical translation.

## 5. Conclusions

ELESW therapy improved glycemic parameters and pancreatic, hepatic, and renal histopathology in T2DM rats, and altered mRNA expression of genes related to *PI3K*/*Akt*/*GLUT4*, *AMPK*, inflammation, pyroptosis, and apoptosis. Direct mechanistic confirmation at the protein level is required. Thus, while ELESW shows potential as a non-pharmaceutical intervention for T2DM, the proposed mechanisms remain to be validated.

## Figures and Tables

**Table 1:** Primer sequences.

Tissue	Gene	Sequence (5′ → 3′)	Amplicon Size/bp
Pancreas	*INS1*	S:TCATAGACCATCAGCAAGCAGG	206
		A:TTCACGACGGGACTTGCGT	
	*INS2*	S:AACAGCACCTTTGTGGTTCTCA	117
		A:GTTGTGCCACTTGTGGGTCC	
	*PDX-1*	S:GAACGCTGGAACAGGGAAGT	198
		A:ACGGGAAAGGGAGATGAACG	
	*GLUT2*	S:GGTCGCCTCGTTCTTTGGT	100
		A:CATCAAGAGGGCTCCAGTCAAC	
Liver	*INSR*	S:GTCGCTCCTATGCTCTGGTGTC	272
		A:GGTCTTCAGGGCAATGTCGTTC	
	*GLUT4*	S:TACTCAGGGCTAACATCAGGGT	234
		A:CTCAGGACAGAAGGGCAACAG	
	*GSK-3β*	S:CACTGTAACATAGTCCGATTGCG	117
		A:TCTGGCGACTCTGTACACTGTTT	
	*GK*	S:AACTTCGTTGGCTCCTCGAC	166
		A:AAAAGCATCGTCCTGCTTGC	
	*PEPCK*	S:GAAAAACACCATCTTCACCAACG	138
		A:GGTTCCTCATCCTGTGGTCTCC	
	*PGC-1α*	S:GAGAAGCGGGAGTCTGAAAGG	219
		A:GTCACAGGTGTAACGGTAGGTAATG	
Kidney	*KIM1*	S:AGGGCTTCTATGTTGGCATGT	188
		A:GATGTTGTCTTCAGCTCGGG	
	*ICAM1*	S:CTCTTGCGAAGACGAGAACCTC	197
		A:CTCGCTCTGGGAACGAATACAC	
	*TGF-β1*	S:GCTGAACCAAGGAGACGGAATA	194
		A:GCAGGTGTTGAGCCCTTTCC	
Housekeeping gene	*β-actin*	S:TGCTATGTTGCCCTAGACTTCG	240
		A:GTTGGCATAGAGGTCTTTACGG	

**Table 2:** Primer sequences.

Gene	Sequence (5′ → 3′)	Amplicon Size/bp
*INSR*	S:GAACAAAGATGACAACGAGGAGTG	152
	A:ACTTACAGATGGTCGGGCAAAC	
*GLUT4*	S:ATCTTTGGCTTCGTGGCATT	166
	A:CCGCAACATACTGGAAACCC	
*AMPK*	S:GGCAGAAGTATGTAGAGCAATCAAA	204
	A:GCAGTCCCTGATTTGGCTTCT	
*GSK-3β*	S:GAGAACTGGTCGCCATCAAGAA	241
	A:TTGACATAAATCACAGGGAGCG	
*GYS2*	S:TGATGTAGTTACTCCAAACGGCT	127
	A:GAGATGACCATAGAAATGACCTCG	
*GCK*	S:CCTGGACAAGCATCAGATGAAAC	212
	A:TCACCATTGCCACCACATCC	
*PEPCK*	S:TTCCCACTGGCATTCGAGATT	234
	A:CCCGCTGAGAAGGAGTTACAATC	
*G6Pase*	S:CCCTGTAACCTGTGAGACTGGAC	105
	A:CCCTGAAAGATGGAAAGAGTAGATG	
*PCNA*	S:AGCCATATTGGAGATGCTGTTG	230
	A:CTGAGTGTCACCGTTGAAGAGAG	
*Caspase-1*	S:TCGCTTTCTGCTCTTCCACA	178
	A:GGCATCTGCGCTCTACCATCT	
*Caspase-3*	S:TGGAAGCGAATCAATGGACTCT	170
	A:TGAATGTTTCCCTGAGGTTTGC	
*ACTIN*	S:CACCCAGCACAATGAAGATCAAGAT	317
	A:CCAGTTTTTAAATCCTGAGTCAAGC	

**Table 3:** Primer sequences.

Gene	Sequence (5′ → 3′)	Amplicon Size/bp
*NLRP3*	S:GCGATCAACAGGAGAGACCTT	124
	A:TCCACTCCTCTTCAATGCTGT	
*GSDMD*	S:AGACACAGAAGGAGGTGGAAGTC	131
	A:GATGGTGACCGTCTTCTTCTGG	

## Data Availability

The raw data supporting the conclusions of this article will be made available by the authors on request.

## Abbreviations

The following abbreviations are used in this manuscript:

T2DM	Type 2 Diabetes Mellitus
ELESW	Electromagnetic Low-Energy Shock Wave
IR	Insulin Resistance
HG	High-glucose damage

## References

[1] American Diabetes Association Professional Practice Committee (2024). Diagnosis and Classification of Diabetes: Standards of Care in Diabetes-2024. Diabetes Care.

[2] Petersen M.C., Shulman G.I. (2018). Mechanisms of Insulin Action and Insulin Resistance. Physiol. Rev..

[3] Cerf M.E. (2013). Beta cell dysfunction and insulin resistance. Front. Endocrinol..

[4] Gerber P.A., Rutter G.A. (2017). The Role of Oxidative Stress and Hypoxia in Pancreatic Beta-Cell Dysfunction in Diabetes Mellitus. Antioxid. Redox Signal..

[5] Muoio D.M., Newgard C.B. (2008). Mechanisms of disease: Molecular and metabolic mechanisms of insulin resistance and beta-cell failure in type 2 diabetes. Nat. Rev. Mol. Cell Biol..

[6] Xie L., Fang B., Zhang C. (2023). The role of ferroptosis in metabolic diseases. Biochim. Biophys. Acta Mol. Cell Res..

[7] Rohm T.V., Meier D.T., Olefsky J.M., Donath M.Y. (2022). Inflammation in obesity, diabetes, and related disorders. Immunity.

[8] American Diabetes Association Professional Practice Committee (2024). 9. Pharmacologic Approaches to Glycemic Treatment: Standards of Care in Diabetes-2024. Diabetes Care.

[9] Huerta T.S., Devarajan A., Tsaava T., Rishi A., Cotero V., Puleo C., Ashe J., Coleman T.R., Chang E.H., Tracey K.J. (2021). Targeted peripheral focused ultrasound stimulation attenuates obesity-induced metabolic and inflammatory dysfunctions. Sci. Rep..

[10] Cotero V., Graf J., Miwa H., Hirschstein Z., Qanud K., Huerta T.S., Tai N., Ding Y., Jimenez-Cowell K., Tomaio J.N. (2022). Stimulation of the hepatoportal nerve plexus with focused ultrasound restores glucose homoeostasis in diabetic mice, rats and swine. Nat. Biomed. Eng..

[11] Ashe J., Graf J., Madhavan R., Wallace K., Cotero V., Abate S., Pandey R.K., Herzog R., Porindla S.N., Shoudy D. (2023). Investigation of liver-targeted peripheral focused ultrasound stimulation (pFUS) and its effect on glucose homeostasis and insulin resistance in type 2 diabetes mellitus: A proof of concept, phase 1 trial. QJM Int. J. Med..

[12] Auersperg V., Trieb K. (2020). Extracorporeal shock wave therapy: An update. EFORT Open Rev..

[13] Chen P.Y., Cheng J.H., Wu Z.S., Chuang Y.C. (2022). New Frontiers of Extracorporeal Shock Wave Medicine in Urology from Bench to Clinical Studies. Biomedicines.

[14] Landscheidt K., Alabdulmohsen A., Hübscher M., Geber B., Hernekamp J.F., Goertz O. (2025). Extracorporeal Shock Waves Therapy for the Treatment of Acute and Chronic Wounds-A Prospective, Monocentric Clinical Trial to Examine the Effect of Shock Waves on Wound Healing. Health Sci. Rep..

[15] Wu K.L., Chiu Y.C., Yao C.C., Tsai C.E., Hu M.L., Kuo C.M., Tai W.C., Chuah S.K., Hsiao C.C. (2019). Effect of extracorporeal low-energy shock wave on diabetic gastroparesis in a rat model. J. Gastroenterol. Hepatol..

[16] Skov-Jeppesen S.M., Yderstraede K.B., Bistrup C., Jensen B.L., Marcussen N., Hanna M., Lund L. (2020). Low-intensity shockwave therapy in the treatment of diabetic nephropathy: A prospective Phase 1 study. Nephrol. Dial. Transplant..

[17] Chen R.F., Lin Y.N., Liu K.F., Lee C.C., Hu C.J., Wang C.T., Wang C.J., Kuo Y.R. (2023). Compare the effectiveness of extracorporeal shockwave and hyperbaric oxygen therapy on enhancing wound healing in a streptozotocin-induced diabetic rodent model. Kaohsiung J. Med. Sci..

[18] Xu L., Zhao Y., Wang M., Song W., Li B., Liu W., Jin X., Zhang H. (2016). Defocused low-energy shock wave activates adipose tissue-derived stem cells in vitro via multiple signaling pathways. Cytotherapy.

[19] Yu G., Guan Y., Liu L., Xing J., Li J., Cheng Q., Liu Z., Bai Z. (2018). The protective effect of low-energy shock wave on testicular ischemia-reperfusion injury is mediated by the PI3K/AKT/NRF2 pathway. Life Sci..

[20] Hsiao C.C., Lin C.C., Hou Y.S., Ko J.Y., Wang C.J. (2019). Low-Energy Extracorporeal Shock Wave Ameliorates Streptozotocin Induced Diabetes and Promotes Pancreatic Beta Cells Regeneration in a Rat Model. Int. J. Mol. Sci..

[21] Mlinar B., Marc J., Janez A., Pfeifer M. (2007). Molecular mechanisms of insulin resistance and associated diseases. Clin. Chim. Acta.

[22] Mao Z.J., Lin M., Zhang X., Qin L.P. (2019). Combined Use of Astragalus Polysaccharide and Berberine Attenuates Insulin Resistance in IR-HepG2 Cells via Regulation of the Gluconeogenesis Signaling Pathway. Front. Pharmacol..

[23] Zhu Y., Zhang H., Wei Y., Cai M., Gu R., Wang Y., Ma Y., Chen L. (2020). Pea-derived peptides, VLP, LLP, VA, and LL, improve insulin resistance in HepG2 cells via activating IRS-1/PI3K/AKT and blocking ROS-mediated p38MAPK signaling. J. Food Biochem..

[24] Chandrasekaran K., Swaminathan K., Chatterjee S., Dey A. (2010). Apoptosis in HepG2 cells exposed to high glucose. Toxicol. In Vitro.

[25] Jiang Q., Yuan Y., Zhou J., Wu Y., Zhou Q., Gui S., Wang Y. (2015). Apoptotic events induced by high glucose in human hepatoma HepG2 cells involve endoplasmic reticulum stress and MAPK’s activation. Mol. Cell Biochem..

[26] Kumar S.M., Swaminathan K., Clemens D.L., Dey A. (2015). GSH protects against oxidative stress and toxicity in VL-17A cells exposed to high glucose. Eur. J. Nutr..

[27] Dolenšek J., Rupnik M.S., Stožer A. (2015). Structural similarities and differences between the human and the mouse pancreas. Islets.

[28] Bechmann L.P., Hannivoort R.A., Gerken G., Hotamisligil G.S., Trauner M., Canbay A. (2012). The interaction of hepatic lipid and glucose metabolism in liver diseases. J. Hepatol..

[29] Alsahli M., Gerich J.E. (2017). Renal glucose metabolism in normal physiological conditions and in diabetes. Diabetes Res. Clin. Pract..

[30] Neelankal John A., Jiang F.X. (2018). An overview of type 2 diabetes and importance of vitamin D3-vitamin D receptor interaction in pancreatic β-cells. J. Diabetes Complicat..

[31] Petersen M.C., Vatner D.F., Shulman G.I. (2017). Regulation of hepatic glucose metabolism in health and disease. Nat. Rev. Endocrinol..

[32] Kanwar Y.S., Sun L., Xie P., Liu F.Y., Chen S. (2011). A glimpse of various pathogenetic mechanisms of diabetic nephropathy. Annu. Rev. Pathol..

[33] Jung C.Y., Yoo T.H. (2022). Pathophysiologic Mechanisms and Potential Biomarkers in Diabetic Kidney Disease. Diabetes Metab. J..

[34] Nguyen Q.M., Srinivasan S.R., Xu J.H., Chen W., Hassig S., Rice J., Berenson G.S. (2011). Elevated liver function enzymes are related to the development of prediabetes and type 2 diabetes in younger adults: The Bogalusa Heart Study. Diabetes Care.

[35] Wu J.G., Kan Y.J., Wu Y.B., Yi J., Chen T.Q., Wu J.Z. (2016). Hepatoprotective effect of ganoderma triterpenoids against oxidative damage induced by tert-butyl hydroperoxide in human hepatic HepG2 cells. Pharm. Biol..

[36] Yaribeygi H., Farrokhi F.R., Butler A.E., Sahebkar A. (2019). Insulin resistance: Review of the underlying molecular mechanisms. J. Cell Physiol..

[37] Guan H., Zhao S., Fang X., Miao R., Zhang Y., Zhang Y., Tian J. (2025). Frontier technologies for investigating endothelial heterogeneity and function in diabetic vascular disease: An updated review. Biomed. Pharmacother..

[38] Papadaki M., Eskin S.G., Ruef J., Runge M.S., McIntire L.V. (1999). Fluid shear stress as a regulator of gene expression in vascular cells: Possible correlations with diabetic abnormalities. Diabetes Res. Clin. Pract..

[39] Boucher J., Kleinridders A., Kahn C.R. (2014). Insulin receptor signaling in normal and insulin-resistant states. Cold Spring Harb. Perspect. Biol..

[40] Hardie D.G., Ross F.A., Hawley S.A. (2012). AMPK: A nutrient and energy sensor that maintains energy homeostasis. Nat. Rev. Mol. Cell Biol..

[41] Foretz M., Guigas B., Viollet B. (2019). Understanding the glucoregulatory mechanisms of metformin in type 2 diabetes mellitus. Nat. Rev. Endocrinol..

[42] Foretz M., Guigas B., Viollet B. (2023). Metformin: Update on mechanisms of action and repurposing potential. Nat. Rev. Endocrinol..

[43] Saltiel A.R., Olefsky J.M. (2017). Inflammatory mechanisms linking obesity and metabolic disease. J. Clin. Investig..

[44] Cheng J.H., Wang C.J. (2015). Biological mechanism of shockwave in bone. Int. J. Surg..

[45] Wang C.J., Cheng J.H., Kuo Y.R., Schaden W., Mittermayr R. (2015). Extracorporeal shockwave therapy in diabetic foot ulcers. Int. J. Surg..

[46] Lin G., Reed-Maldonado A.B., Wang B., Lee Y.C., Zhou J., Lu Z., Wang G., Banie L., Lue T.F. (2017). In Situ Activation of Penile Progenitor Cells With Low-Intensity Extracorporeal Shockwave Therapy. J. Sex. Med..

[47] Shi C., Wang Q., Rao Z., Shi Y., Wei S., Wang H., Lu X., Wang P., Lu L., Zhou H. (2020). Diabetes induces hepatocyte pyroptosis by promoting oxidative stress-mediated NLRP3 inflammasome activation during liver ischaemia and reperfusion injury. Ann. Transl. Med..

[48] Zhang X., Yan X., Wang C., Tang T., Chai Y. (2014). The dose-effect relationship in extracorporeal shock wave therapy: The optimal parameter for extracorporeal shock wave therapy. J. Surg. Res..

[49] Takahashi T., Nakagawa K., Tada S., Tsukamoto A. (2019). Low-energy shock waves evoke intracellular Ca^2+^ increases independently of sonoporation. Sci. Rep..

[50] Orrenius S., Zhivotovsky B., Nicotera P. (2003). Regulation of cell death: The calcium-apoptosis link. Nat. Rev. Mol. Cell Biol..

[51] Varghese E., Samuel S.M., Sadiq Z., Kubatka P., Liskova A., Benacka J., Pazinka P., Kruzliak P., Büsselberg D. (2019). Anti-Cancer Agents in Proliferation and Cell Death: The Calcium Connection. Int. J. Mol. Sci..

[52] McCormack J.G., Denton R.M. (1990). Intracellular calcium ions and intramitochondrial Ca^2+^ in the regulation of energy metabolism in mammalian tissues. Proc. Nutr. Soc..

[53] Lian L., Ye X., Wang Z., Li J., Wang J., Chen L., Reinach P.S., Ma X., Chen W., Zheng Q. (2025). Hyperosmotic stress-induced NLRP3 inflammasome activation via the mechanosensitive PIEZO1 channel in dry eye corneal epithelium. Ocul. Surf..

